# Accurate cytogenetic biodosimetry through automated dicentric chromosome curation and metaphase cell selection

**DOI:** 10.12688/f1000research.12226.1

**Published:** 2017-08-09

**Authors:** Jin Liu, Yanxin Li, Ruth Wilkins, Farrah Flegal, Joan H.M. Knoll, Peter K. Rogan

**Affiliations:** 1 Department of Biochemistry, Schulich School of Medicine and Dentistry, University of Western Ontario, London, ON, N6A 5C1, Canada; 2 Cytognomix Inc., London, ON, N5X 3X5, Canada; 3 Consumer and Clinical Radiation Protection Bureau, Health Canada, Ottawa, ON, K1A 1C1, Canada; 4 Canadian Nuclear Laboratories Radiobiology & Health, Chalk River, ON, K0J 1J0, Canada; 5 Department of Pathology and Laboratory Medicine, Schulich School of Medicine and Dentistry, University of Western Ontario, London, ON, N6A 5C1, Canada

**Keywords:** Ionizing Radiation, Radiation Exposure, Computer-Assisted Image Processing, Software, Quality Control, Cytogenetics, Metaphase, Mass casualty incidents

## Abstract

Accurate digital image analysis of abnormal microscopic structures relies on high quality images and on minimizing the rates of false positive (FP) and negative objects in images. Cytogenetic biodosimetry detects dicentric chromosomes (DCs) that arise from exposure to ionizing radiation, and determines radiation dose received based on DC frequency. Improvements in automated DC recognition increase the accuracy of dose estimates by reclassifying FP DCs as monocentric chromosomes or chromosome fragments. We also present image segmentation methods to rank high quality digital metaphase images and eliminate suboptimal metaphase cells. A set of chromosome morphology segmentation methods selectively filtered out FP DCs arising primarily from sister chromatid separation, chromosome fragmentation, and cellular debris. This reduced FPs by an average of 55% and was highly specific to these abnormal structures (≥97.7%) in three samples. Additional filters selectively removed images with incomplete, highly overlapped, or missing metaphase cells, or with poor overall chromosome morphologies that increased FP rates. Image selection is optimized and FP DCs are minimized by combining multiple feature based segmentation filters and a novel image sorting procedure based on the known distribution of chromosome lengths. Applying the same image segmentation filtering procedures to both calibration and test samples reduced the average dose estimation error from 0.4 Gy to <0.2 Gy, obviating the need to first manually review these images. This reliable and scalable solution enables batch processing for multiple samples of unknown dose, and meets current requirements for triage radiation biodosimetry of high quality metaphase cell preparations.

## Abbreviations

ADCI, Automated Dicentric Chromosome Identifier and dose estimator; CNL, Canadian Nuclear Laboratories; DC, Dicentric chromosome; DCA, Dicentric chromosome assay; FP, False positive; HC, Health Canada; K–S, Kolmogorov–Smirnov test; MC, Monocentric chromosome; MC-DC SVM, Monocentric-Dicentric Support Vector Machine; ML, Machine learning; SCS, Sister chromatid separation; SD, Standard deviation; SVM, Support Vector Machine; TP, True positive.

## Introduction

Analysis of microscopy images of metaphase cells demonstrates the damaging effects of ionizing radiation and can be used to measure the amount of radiation absorbed. The gold standard method for radiation biodosimetry, the dicentric chromosome assay (DCA), uses the frequency of aberrant dicentric chromosomes (DCs) formed after radiation exposure to determine the dose received by an individual (in Gy). While some aspects of the assay have been successfully streamlined, the overall throughput remains limited by the labour-intensive identification of DCs in many cells. This affects the timely estimation of radiation exposure, especially for testing multiple affected individuals in a large accident or a mass casualty nuclear event ^1, 2^.

The selection of images of adequate quality for accurate identification of the chromosome damage is a prerequisite to automating DCA. The decision to select or exclude particular microscope images based on the quality of metaphase cells has been performed manually, which is impractical given the increasing sizes of datasets produced by automated image capture systems. Image quality assessment has traditionally compared new data relative to reference images ^3^, complex mathematical models ^4^, or distortions from a training set recognized by machine learning ^5^. Such generic approaches are not appropriate in the DCA because features tailored for ranking morphologically diverse chromosome images are not easily generalized as entropic or other measures applying frequency filters to intensity distributions. We demonstrate that quality chromosomal images can be selected for the DCA using supervised, image segmentation rules aimed at categorizing the preferred images and eliminating false positive (FP) DCs.

**Figure 1.**
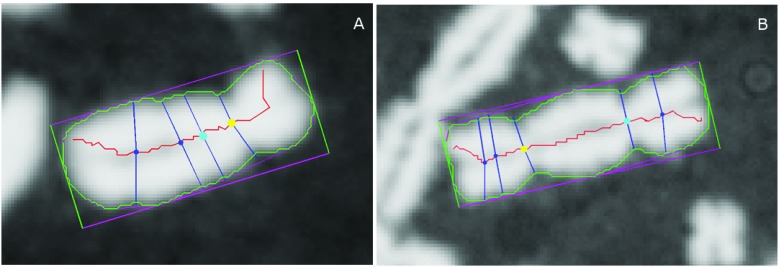
Chromosome images processed by ADCI, annotated with key segmentation features. ( **A**) Monocentric and ( **B**) Dicentric chromosome. Chromosome contour overlaid in green, long-axis centreline in red. For reference, the minimum bounding box of the contour is also displayed in magenta and green. Yellow and cyan markers on the centerline indicate the top-ranked and 2 ^nd^-ranked centromere candidates, respectively, and all other candidates are indicated with a dark blue marker. For each centromere candidate, their corresponding width traceline (crossing through the candidate and running approximately orthogonal to the centerline) are displayed in dark blue. The arc lengths of width tracelines running down the centerline (not all shown) are used to construct a chromosomal width profile. Note that for the monocentric chromosome ( **A**), the top-ranked candidate correctly labels the true centromere location, while the 2 ^nd^-ranked candidate labels a minor non-centromeric constriction. Meanwhile, for the dicentric example ( **B**), both the top and 2 ^nd^-ranked candidates label true centromere locations. By comparing features extracted from the top 2 candidates (including width and pixel intensity information), the software will determine if the chromosome is monocentric or dicentric.

We previously developed the Automated Dicentric Chromosome Identifier and Dose Estimator (ADCI) software to automate DC detection and estimate radiation exposures ^6– 11^. Briefly, ADCI uses image segmentation techniques to extract possible chromosomes. Preprocessing image filters remove most but not all non-chromosomal objects (e.g. debris, nuclei, overlapping chromosomes). Each remaining object is regarded as a single, intact, post-replication “chromosome-like” object. Each of these objects is processed by a series of algorithms ^7– 10^ which create a quantitative profile measuring chromosome width from one telomere to the other. Potential centromere locations (“centromere candidates”) are identified at constrictions in the width profile ( Figure 1) ^12^. Machine learning (ML) modules then use features sourced from computer vision analysis of each chromosome to classify centromeres and dicentric chromosomes ^6, 11^. An initial Support Vector Machine (SVM) ranks potential centromere candidates in each chromosome according to their corresponding distances to the hyperplane that distinguishes centromeres from non-centromeric constrictions; then, another SVM scores the chromosome as either monocentric (MC) or dicentric (DC), using features derived from the top two centromere candidates.

Samples from blood exposed *ex vivo* to known radiation doses are processed by ADCI to construct a dose-response calibration curve. The average frequency of DCs per cell in dose calibrated samples, i.e. the radiation response, is fit to a linear-quadratic function. Responses for test samples exposed to unknown radiation levels can then be analyzed with this function to estimate the corresponding doses.

We noticed that metaphase cell images of inconsistent, lower quality can affect the accuracy of dose estimation by ADCI. Previous studies evaluated the efficacy of ADCI at chromosome classification and dose estimation ^10, 11^. While the sensitivity (recall) for DCs was acceptable (~70%) and relatively constant at all radiation exposure levels, precision showed a strong dependence on dose. Chromosome misclassification, in particular FPs comprised a larger fraction of DCs at low (≤1 Gy) relative to high (3–4 Gy) doses; at 1 Gy, FPs could outnumber true positive (TP) dicentrics by a factor of 4 to 5. Consequently, ADCI-processed samples exhibited a reduced range of accurate responses to radiation compared to manually scored samples. Although use of the same algorithm to derive the calibration curve compensates for some of these differences, reliability of the dose estimation ultimately hinges on DC classification accuracy. As DCs are always greatly outnumbered by MCs in a cell (background frequency in normal, unexposed individuals is one DC per 1000 cells ^6^), this study focuses on improving the distinction between TP and FP DCs without compromising sensitivity.

FPs reflect inadequacies in interpreting certain chromosome morphologies or non-chromosomal objects as DCs. To improve overall DC classification accuracy, FPs must be selectively identified and removed without limiting TP counts. We first investigated FPs to categorize problematic cases and devised a set of post-processing object segmentation filters to eliminate them. Then, to ensure consistent overall performance within a set of images from a sample, statistical filters were developed to remove poor quality cells. Frequently, these images either lacked any chromosomes or contained incomplete metaphase cells, misclassified interphase or micro-nuclei as metaphase cells, or incorrectly segmented sister chromatids as individual chromosomes. Chromatid separation and chromosome fragments increase the object count in an image, but the pixel areas of said objects are smaller than actual chromosomes. Chromosome-overlaps reduce the object count, but their areas tend to exceed those of discrete chromosomes. Each proposed statistical filter was tested individually, and the best performing filters were applied cumulatively, then tested on cytogenetic dosimetry data at various radiation exposures. Effects of these filters on classification performance and dose estimation were then evaluated with dose-blinded, irradiated samples obtained from biodosimetry laboratories at Health Canada (HC) and Canadian Nuclear Laboratories (CNL).

This hybrid approach selects images based on optimal metaphase cell image properties and customized segmentation, and by identification and elimination of FP DCs. These improvements in ADCI ensure timely, reproducible, and accurate quantitative assessment of acute radiation exposure.

## Methods

Cytogenetic image data were obtained at biodosimetry laboratories at HC and CNL, according to International Atomic Energy Agency (IAEA) guidelines. Blood samples were irradiated by an XRAD-320 (Precision X-ray, North Branford, CT) at Health Canada and processed at both laboratories. Samples were obtained with written informed consent from anonymous donors by the HC laboratory as approved by the Health Canada and Public Health Agency of Canada’s Research Ethics Board of protocol: “Development of Biological Dosimeters for Ionizing Radiation.” Peripheral blood lymphocyte samples were cultured, fixed, and stained at each facility according to established protocols ^2, 12^. Metaphase images from Giemsa-stained slides were captured independently by each laboratory using an automated microscopy system (Metasystems, Newton, MA). One set of metaphase images from CNL and two sets from HC ( Table 1) were used for development and initial testing of the proposed algorithms. After image processing by ADCI, the identified DCs were manually reviewed and of the numbers of TPs or FPs were tallied. Calibration curves were prepared based on 6 samples of known radiation dose ( Table 2). An additional 6 samples ^11^ were initially blinded to the actual radiation exposures as test samples ( Table 3). Test samples were exposed to a range of radiation doses bounded by the doses of samples used to construct the calibration curve. The sample naming convention is the laboratory name followed by the sample identifier, e.g. HC1Gy signifies the 1 Gy calibration sample prepared at HC, whereas CNL-INTC03S04 represents the test sample, INTC03S04, from an international laboratory inter-comparison exercise that was prepared at CNL (which had been exposed to 1.8 Gy).

**Table 1.** Metaphase image sets used in development and validation of DC filters.

Dataset name	HC-mixed *		
	*HC-low*	*HC-high*	*CNL-low*
**Lab source**	Health Canada	Health Canada	Canadian Nuclear Laboratories
**Radiation dose (Gy)**	1	3-4	1
**No. of images**	198	216	256
**No. of** chromosomes **	8041	8697	10583
**No. of TPs**	20	163	14
**No. of FPs**	97	61	82

* *HC-mixed* refers to a combined set of all images from both the *HC-low* + *HC-high* datasets

**Defined as number of valid segmented objects defined by ADCI.

**Table 2.** Metaphase image samples used in construction of dose calibration curves.

Sample (HC or CNL)	Physical dose (Gy)	No. of images, HC	No. of images, CNL
0Gy	0	731	798
0.5Gy	0.5	1054	1532
1Gy	1	1566	841
2Gy	2	1147	996
3Gy	3	1212	1188
4Gy	4	909	1635
5Gy	5	1019	-

**Table 3.** Metaphase image samples used in evaluation of dose assessment performance.

Sample name	Physical dose (Gy)	No. of images, HC preparation	No. of images, CNL preparation
INTC03S01	3.1	540	500
INTC03S08	2.3	637	500
INTC03S10	1.4	708	n/a
INTC03S04	1.8	600	957
INTC03S05	2.8	1136	1527
INTC03S07	3.4	477	735

n/a: data were not available.

Each calibration and test sample consisted of images from the same individual. HC provided an unselected set of all metaphase cells that were automatically recognized and captured using the default classifier of the microscopy system. By contrast, CNL previously manually curated a set of 500 high quality metaphase cell images, selected according to IAEA guidelines ^12^, which deem metaphase cells analyzable based on chromosome count, distribution and morphology.

### 1) ADCI settings and metaphase image data

ADCI software (V1.0) ^11^ was used for DC detection and dose prediction, setting the tuning parameter, σ, for the MC-DC SVM to 1.5. Software libraries were initially developed as available MATLAB scripts to test segmentation filters that detected FP DCs; once validated, C++ versions of these libraries were integrated into ADCI. For validation, two low dose and one high dose dataset were used ( Table 1; the combination from HC comprise the *HC-mixed* image set).

### 2) Filtering out false positive objects

Quantitative morphological filters to delineate FP DCs were created and tested (i-viii, below). Each filter is designed to detect one or more of 6 FP morphological subclasses of FPs (described in Supplementary File 1). The FPs result from either I) excessive sister chromatid separation (SCS), II) fragmented or III) overlapping chromosomes, IV) chromosomes with highly variable boundaries or contours, V) non-chromosomal cellular debris, or VI) errors in the machine learning algorithms that detect centromere candidates and distinguish MCs from DCs.

**Figure 2.**
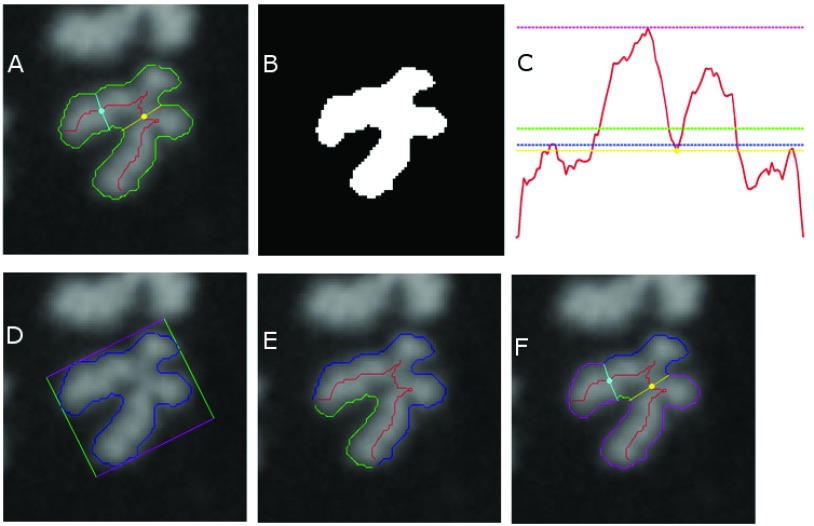
A visualization of DC filter scores for a particular false positive (FP). *DC filters are defined in Methods*. ( **A**) A processed FP (chromosome with SCS), with contour in green, centerline in red, top-ranked centromere candidate and its width traceline in yellow, 2 ^nd^-ranked centromere candidate and its width traceline in cyan. ( **B**) Filter i: Thresholded binary image of the chromosome is used to calculate pixel area (in white). ( **C**) Filters ii–v: Width profile along centerline is shown in red (horizontal axis plots centerline location, vertical axis plots width), with mean width in green (filter ii), median width in blue (filter iii), max width in magenta (filter iv), and width of top centromere candidate in yellow (filter v). ( **D**) Filter vi: Contour in blue and its minimum bounding rectangle in magenta and green. ( **E**) Filter vii: Partitioning of contour at centerline endpoints (intersection of red line with contour) into two segments, green and blue. ( **F**) Filter VIII: Traceline endpoints of top 2 centromere candidates (intersection of yellow and cyan lines with contour) are used to partition contour into 4 segments (1 blue, 1 green, 2 magenta); relative arc lengths of blue and green segments are taken into consideration.

The set of N chromosomes in any metaphase image is denoted by *{c _1_,…,c _N_}* and c* denotes the predicted DC of interest. Each filter (designated i – viii, below) classifies c* as either a TP or FP by comparing its filter score against a heuristically-defined threshold that is independent of laboratory source. Quantitative thresholds were established for each filter to eliminate the maximum number of FPs, without compromising detection of TP. Due to the relatively low frequency of DCs in the samples, maximal detection of TPs is essential for accurate dose estimation. Since FPs generally produce lower filter scores than TPs (i.e. lower area, lower width, less oblong footprint, more asymmetrical), FPs were selected by eliminating candidate DCs with scores below each threshold. The corresponding FP filter scores were calculated for all DCs in the *HC-mixed* image set ( Table 1), and a heuristic threshold (to 2 significant digits; see below) was set to the minimum value observed in TPs for each filter. Thresholds for filters vi, vii and viii were calculated by repeating the same procedure on a set of 244 TP chromosomes from the MC-DC SVM training set ^6^, and the final thresholds were set to the lower of each pair of values.

i. **Area filter:** *A(c)* denotes the pixel area occupied by chromosome *c* ( Figure 2B). c* was classified as FP, if A(c*)/median({A(c _1_),…,A(c _N_)}) < 0.74 or as TP otherwise. This filter targets small chromosomes commonly displaying SCS ( Figure 2A) and chromosome fragments.
ii. **Mean width filter:** *W _mean_(c)* denotes the mean value of the width profile of chromosome *c* ( Figure 2C). c* was classified as FP if W _mean_(c*)/median({W _mean_(c _1_),…, W _mean_(c _N_)}) < 0.80 or as TP otherwise. This filter targets SCS and chromosome fragments.
iii. **Median width filter:** *W _med_(c)* denotes the median value of the width profile of chromosome *c* ( Figure 2C). c* was classified as FP if W _med_(c*)/median({W _med_(c _1_),…, W _med_(c _N_)}) < 0.77, or as TP otherwise. This filter targets SCS and chromosome fragments.
iv. **Max width filter:** *W _max_(c)* denotes the maximum value of the width profile of chromosome *c* ( Figure 2C). c* was classified as FP if W _max_(c*)/median({W _max_(c _1_),…, W _max_(c _N_)}) < 0.83, or as TP otherwise. This filter targets SCS and chromosome fragments.
v. **Centromere width filter:** *W _cent_(c)* denotes the width of chromosome *c* at the position of the top-ranked centromere candidate ( Figure 2C). c* was classified as FP if W _cent_(c*)/median({W _cent_(c _1_),…,W _cent_(c _N_)}) < 0.72, or as TP otherwise. This filter targets SCS and chromosome fragments.
vi. **Oblongness filter:** *S(c)* denotes the pair of side lengths of the minimum bounding rectangle enclosing the contour of chromosome *c* ( Figure 2D). c* was classified as FP if 1 − min(S(c*))/max(S(c*)) < 0.28, or as TP otherwise. This filter targets acrocentric chromosomes with SCS and some cases of overlapping chromosomes.
vii. **Contour symmetry filter:** *L(c)* denotes the pair of arc lengths of contour halves produced by partitioning the contour of chromosome *c* at its centerline endpoints ( Figure 2E). c* was classified as FP if min(L(c*))/max(L(c*)) < 0.51, or as TP otherwise. This filter targets SCS.
viii. **Intercandidate contour symmetry filter:** *L _C_(c)* denotes the pair of arc lengths of the contour regions of chromosome *c* that run between the traceline endpoints of its top 2 centromere candidates ( Figure 2F). c* was classified as FP if min(L _C_(c*))/max(L _C_(c*)) < 0.42, or as TP otherwise. This filter targets SCS and some instances of overlapping chromosomes.

**Determination of optimal filter subset:** The same chromosome segmentation features were present in different segmentation filters, usually in combination with other elements (i.e. width for filters ii–v, contour symmetry for vi–viii) and/or targeted the same morphological subclass (notably, SCS). Thus, the “optimal” filter subset (termed “FP filters”) was defined as the subset of filters that maximized reclassification of the maximum number of FPs, while minimizing redundant detection of the same FPs. The performance for a given set of filters was the cumulative percentage of FPs removed by any of its filters, based on the *HC-mixed* set of images ( Table 1). Using a forward selection approach, individual filters were added iteratively to identify those that produced the largest improvement in performance.

**Figure 3.**
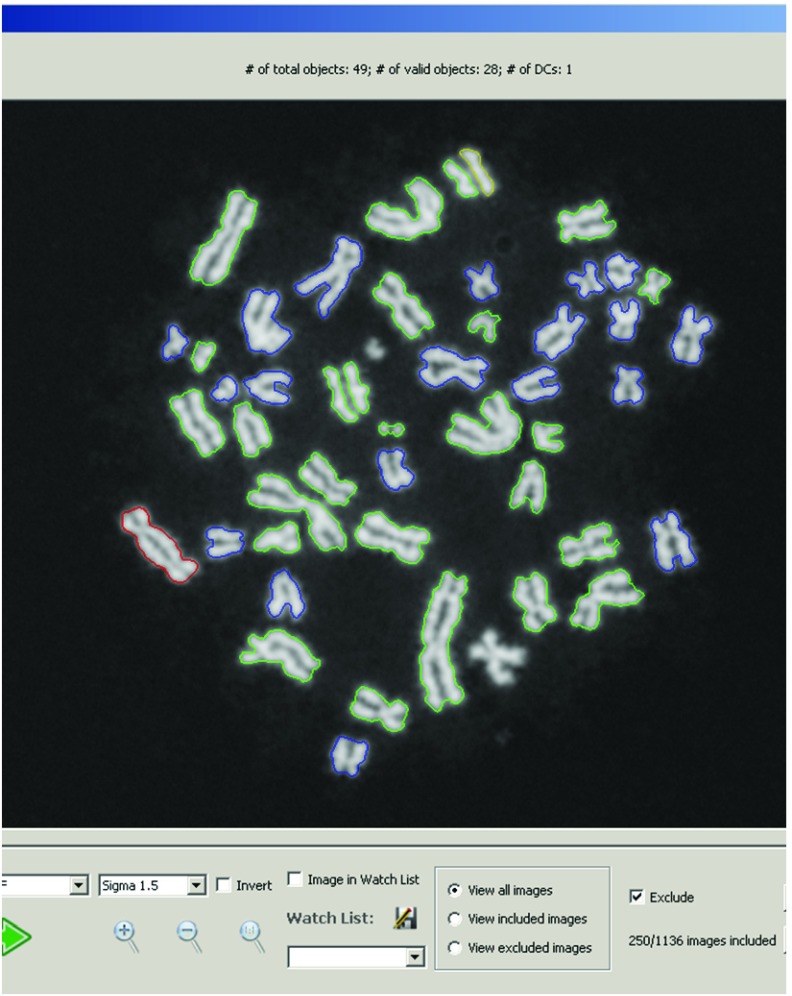
Cell image viewer in ADCI demonstrating example of a corrected false positive (FP) DC. Graphical User Interface for viewing cell images within a sample processed by ADCI ^11^. Valid segmented objects (generally chromosomes, but occasionally nuclei or debris) are shown with coloured contours. Red contours indicate predicted DCs, yellow contours indicate chromosomes that were initially classified as DC and then reclassified by the FP filters (example at 12 o’clock), green contours indicate predicted MCs, and blue contours indicate objects that could not be further processed after image segmentation. Below the cell image, options were added to allow manual inclusion or exclusion of images within a sample from dose determination.

**Modifications to ADCI:** After chromosome processing and MC-DC SVM classification ^11^ but prior to dose determination, all DC chromosomes inferred by ADCI were analyzed with the FP filters. DCs classified as FPs by any of the filters were reclassified and the remaining TP DCs were used for dose determination. The contours of DCs that were reclassified as MCs are outlined in yellow in the ADCI metaphase image viewer ^11^ ( Figure 3; top centre).

### 3) Dose estimation analysis

In ADCI, a pre-computed dose-response calibration curve is also used to estimate radiation absorbed in samples with unknown whole body exposures ^11^. For a given sample, the radiation response is the ratio of the number of DCs detected to the number of selected metaphase cells. Calibration curves can be generated either from a set of samples of known exposures either by determining the response for each sample automatically with ADCI, or by entering the corresponding response from manually scored samples, and fitting the dose-response paired data to a linear-quadratic curve by regression. Because sample preparation protocols can vary and affect results, dose estimation of test samples (of unknown exposures) were performed with calibration curves generated with data from the same laboratory ^11^.

The impact of segmentation filters to remove FPs on calibration curves was determined for the 0, 0.5, 1, 2, 3 and 4 Gy calibration samples. Radiation doses were estimated for CNL and HC test samples using the HC calibration curve after applying the same FP filters ( Table 6).

### 4) Effect of filtering on manually image selected HC data

We compared HC calibration curves derived from manually curated samples with the FP filters either enabled or disabled to assess the impact of image selection on dose accuracy ( Table 2). The criteria for manually curated HC samples were similar to the manual image selection performed by CNL. These images required: A) a complete complement of approximately 46 chromosomes, >40 segmented objects, <5 segmented objects from different nuclei if multiple nuclei present; B) exclusion of metaphase cells with “harlequin” hemi-stained chromosomes (indicative of multiple rounds of division after radiation exposure) that distort true DC frequencies ^10^; C) images with <5 incorrectly-segmented chromosomes (chromosome overlaps indicating poor spreading), fragments (indicating sister chromatid separation) and overly-noisy contours (indicating poor image contrast); and D) an adequate degree of chromosome condensation. Depending on the stage of metaphase arrest, the degree of chromosome condensation can differ ^1, 13^. Prometaphase cells have longer chromosomes, are less rigid, exhibit greater overlap and less well-defined centromere constrictions, all of which pose significant challenges for automated chromosome classifiers ^1, 14^. Metaphase images with longer, thinner chromosomes (roughly corresponding to >550-band level ^14^) were also excluded.

A minimum sample size of 500 cells per dose was adopted from IAEA recommendations ^12^. Cell images selected from HC samples with automatic morphology filtering (see Methods section #5) were compared with a high quality set of images that were manually identified using the ADCI microscope viewer. For each sample, consecutive images meeting all criteria were evaluated manually until a sufficient number of cells were accrued. DC classifications were hidden during image selection to minimize bias. After generation of the curated HC calibration curves, the radiation doses of the three HC test samples ( Table 3) were re-estimated on the new curves, with and without the FP filters enabled.

### 5) Automated removal of suboptimal images by morphology filtering

Manual selection of images assures consistency and reliability of metaphase data, which increases accuracy in DC analysis. Exclusion of lower quality images was automated in ADCI, since it was expected to reduce the number of FP DCs, thereby more accurately estimating radiation exposures.

We derived a set of image selection filters, implemented as available Python scripts, by segmenting features (I-VI, below) that eliminate metaphase cells in a sample with characteristics that increased the number of FPs:

I. **Length-width ratio filter (LW)** is based on the average length-width ratio of all chromosomes in an image. For a given chromosome *c* in a given image *I* containing *N* chromosomes, *L(c,I)* denotes the arc length of the centerline of *c*, *W _mean_(c,I)* denotes the mean value of the width profile of *c, SD* is the standard deviation on *W _mean_(c,I)*, and *T* denotes the threshold value of SD common to all of these filters that distinguishes acceptable from outlier images. *MW(I)* is defined as *mean{L(c _1_,I)/W _mean_(c _1_,I),…,L(c _N_,I)/W _mean_(c _N_,I)}*. I* is removed if MW(I*) > mean{MW(I _1_),…,MW(I _M_)} + T×SD{MW(I _1_),…,MW(I _M_)}.
II. **Centromere candidate density filter (CD)** counts occurrences of centromere candidates in chromosomes and eliminates images containing chromosomes with a high density of candidate centromeres. For a given chromosome *c* in image *I* containing *N* chromosomes, *L(c,I)* denotes the arc length of the centerline of *c*, and *N _cent_(c,I)* denotes the number of centromere candidates along *c*. *CD(I)* is defined as the *mean{N _cent_(c _1_,I)/L(c _1_,I),…,N _cent_(c _N_,I)/L(c _N_,I)}*. I* is removed if CD(I*) > mean{CD(I _1_),…,CD(I _M_)} + T×SD{CD(I _1_),…,CD(I _M_)}.
III. **Contour finite difference filter (FD)** represents the smoothness of contours of segmented objects in an image. It eliminates images with non-chromosomal objects with smooth contours, such as nuclei or micronuclei. For a given chromosome *c* in a given image *I* containing *N* chromosomes, *WP _D_(c,I)* denotes the set of first differences of the normalized width profile of *c* (range normalized to interval [0,1]). *WD(I)* is defined as the *mean{mean{abs{WP _D_(c _1_,I)}},…,mean{abs{WP _D_(c _N_,I)}}}*. I* is removed if WD(I*) < mean{WD(I _1_),…,WD(I _M_)} – T×SD{WD(I _1_),…,WD(I _M_)}.
IV. **Total object count (ObjCount) filter** is based on the number of all objects detected in an image. Values lying outside of a threshold range are rejected to eliminate images with multiple metaphases or excessive cellular debris. Based on empirical analyses, the suggested object count range falls within the interval [40, 60].
V. **Segmented object count (SegObjCount) filter** is based on the number of objects processed by the gradient vector flow ^7^ (GVF) algorithm in an image. It is applied in the same way as filter IV. The suggested range for the object count interval is [35, 50].
VI. **Classified object ratio (ClassifiedRatio) filter** is derived from the ratio of objects recognized as chromosomes to the total number of segmented objects. It excludes images in which ADCI fails to process the majority of chromosomes. An image is removed if the ratio is less than either 0.6 or 0.7, which is determined by the desired level of stringency for this filter.

Filters I and II detect cells in prometaphase (having relatively long and thin chromosomes), with prominent sister chromosome separation, and with highly bent and twisted chromosomes. Filter III detects overly-smooth contours characterized by images containing intact nuclei and otherwise incomplete chromosome sets. The total object count (IV) and segmented object count filters V enrich for nearly normal metaphase images of approximately 46 chromosomes. These filters are then used to exclude images with extreme object counts. Filter VI selects images based on effectiveness of chromosome recognition by ADCI.

Image level filters I-III are based on the z-scores of different properties and comprise all objects in an image. For metaphase image I* in a sample containing *M* images, *{I _1_,...,I _M_}*, *{c _1_,…,c _N_}* denotes the set of *N* chromosomes within image I*. This SD value was determined to be 1.5 by varying *T* and applying these filters to the HC 2Gy calibration sample ( Table 2). The corresponding thresholds for filters IV-VI were also derived from testing multiple samples.

**Image ranking by combining image selection filters:** Applying these filters sequentially to the same image distinguished the metaphase images used for dose estimation from lower quality images. Features consisting of counts, ratios and Z-scores for image filters I-VI were linearly combined to globally assess image quality. The combined score is one representation of the degree to which a particular image deviates from the population in a sample:

*Combined Z Score* *=w(LW)×z(LW)+w(CD)×z(CD) – w(FD)* × *z(FD)*+ *w(ObjCount)*×| *z(ObjCount)* | + *w(SegObjCount)*×| *z(SegObjCount)* |- *w(ClassifiedRatio)*× *z(ClassifiedRatio)*

Smaller Combined Z Scores represent higher quality images. Longer and thinner chromosomes in the image will increase the LW score, whereas bent and twisted chromosomes increase the CD term. Decreased chromosome concavity results in a higher FD score. The object and segmented object counts and their respective Z scores are related to chromosome distribution, and the level of sister chromatid separation in an image. These terms contribute to higher Combined Z Scores for images exhibiting either incomplete cells, multiple cells or severe sister chromatid separation. The Classified Ratio terms produce high scores for images that the algorithm does not process accurately. Each feature has a positive free parameter, weight, to adjust its contribution to the total score. Weights are determined by evaluating many possible weights using a grid search technique, and selecting those that minimize the error in curve calibration. The optimal weights for calibration samples are expected to perform similarly on test samples exposed to unknown radiation levels, assuming that the calibration and test samples have comparable chromosome morphologies. The Combined Z Score, however, cannot be used to compare the overall qualities of different samples, as Z-scores are normalized within each sample.

**Image comparisons based on chromosome length distributions:** The previously described tests use image morphology as the primary consideration in assessing metaphase image quality. The most common problems in lower quality metaphase cells are severe sister chromatid separation, excessive chromosome overlap, fragments of chromosomes in image segmentation, and multiple cells or incomplete cells in the same image. These often result in changes in either the number or the sizes of segmented objects. These tests do not account for the known relationships between the chromosomes in a cell with a nearly normal karyotype.

We derived a novel quality measure based on the observation that lengths and areas of chromosome images (in pixels) are approximately proportional to the well-known base-pair counts for each human chromosome. By comparing the distribution of observed chromosome object lengths with this “gold standard” inferred from the lengths of chromosomes in the reference human genome sequence, the overall quality of chromosome segmentation can be assessed in each cell image. Excluding chromosome abnormalities, which result from radiation exposure and are randomly distributed among cells, individual chromosome lengths are approximated by their corresponding chromosome areas (in pixels), since the actual chromosome lengths are difficult to measure accurately. Once noisy non-chromosomal objects, nuclei and large overlapped chromosome clusters have been removed, the areas of each remaining object are then determined relative to the total area of all chromosomes. The chromosomes in a metaphase cell are binned into three groups corresponding to the ISCN cytogenetic classification system ^16^: The (AB) set comprises the A and B chromosome groups, (C) contains all of group C, and (DG) includes the D, E, F, and G groups. A single chromosome in group AB contains > 2.9% of nucleotides in the complete genome (determined by the shortest B group chromosome). A chromosome in category C has < 2.9% (determined by the longest C group chromosome), but > 2% (determined by the shortest C group chromosome) of nucleotides in the complete genome. Any chromosome in category DG contains < 2% of the complete genome (determined by the longest D group chromosome). These thresholds, 2.9% and 2% of the genome length, are respectively considered to be the maximum lengths of X and Y chromosomes. These thresholds are then applied to the areas of each chromosome object to count the number of chromosomes in each category in a metaphase image. An ideal metaphase image will have 10 AB chromosomes, 16 C chromosomes and 20 DG chromosomes in a female, and 10 AB chromosomes, 15 C chromosomes and 21 DG chromosomes in a male. We find that images with many overlapping chromosomes will have increased AB chromosome counts, while images with excessive sister chromatid separation generally have elevated DG chromosome counts. The quality of a metaphase image is determined by comparing the observed quantities of chromosomes in each group to the female or male standard. In practice, the result for an image is treated as a 3-element vector (AB, C, DG) and the Euclidean distance between the observed vector and the ideal standard is determined. Larger group bin distances correspond to less satisfactory images. We find that this measure appears to be universally applicable to metaphase images from different samples.

Sorting all images in a sample by either their Combined Z Score or by chromosome area Group Bin distance ranks cells according to metaphase quality for subsequent DC analyses. Image selection models can also be created in multiple stages by first qualifying images with chromosome morphology filters and then by selecting the top scoring images according to their Combined Z Scores or Group Bin distances.

### 6) Sample quality confidence measurement

Cytogenetic artifacts, such as sister chromatid separation and chromosome fragmentation, interfere with correct identification of DCs, thereby compromising reliability of dose estimates. This motivated the development of criteria to evaluate how well automated cell and FP curation improves sample quality. Samples exposed to low energy transfer, whole-body irradiation exhibit DC distributions that follow a Poisson distribution ^17^ in all cells. The number of DC occurrences in a cell is constructed as a probability model of a sample. Each DC is assumed to be independent of other DCs in the first cell division and the rate at which DCs occur is constant for a single sample at a given radiation dose. The DC distribution detected either manually or by ADCI can be approximated by the Poisson statistic, with the λ parameter corresponding to the average number of DCs per cell in a sample.

Deviation from the Poisson distribution can occur when either some TPs are not accounted for or when FP DCs have not been reclassified. We evaluated post-processing sample quality by comparing the observed distribution of DCs in each sample (manual and automated) to its corresponding Poisson distribution. Observed and Poisson DC distributions were analyzed with the Pearson chi-squared goodness-of-fit test, which indicates the likelihood of rejecting the null hypothesis that the DCs were Poisson distributed. Only samples with ≥ 1 DC were analyzed. Very low p-values at or below α = 0.005 (99.5% confidence level) reject the null hypothesis and indicate lower quality samples.

## Results

### Application of chromosome morphology filters to remove FPs

False positive DCs (n=98) from a set of metaphase cells exposed to low dose radiation were classified into morphological subclasses to identify and ultimately eliminate these objects (described in Supplementary File 1). FP subclasses ( Figure S1; subclasses A–F) included those exhibiting high levels of sister chromatid separation (A, n=51), chromosome fragmentation (B, n=10), overlap (C, n=17), noisy contour (D, n=5), cellular debris (E, n=4), as well as inaccurate recognition by either the centromere candidate ^10^ or MC/DC ^6^ machine learning algorithms (F, n=11).

Segmentation filters i–viii were applied to reclassify FPs in these images. Scale-invariant filters were tested to determine thresholds that selectively removed subclasses I-III without eliminating any TPs. Of the 51 SCS cases, 35 involved short, acrocentric chromosomes. FPs were distinguished from TPs based on either their lower relative pixel area or width (filters i–v), substantially non-oblong footprint (filter vi), or substantial contour asymmetry across the centerline (filters vii and viii). For filters i-v, normalization to median scores of other objects in the same image was performed, as well as normalization to other measures of central tendency (e.g. z-score, mean, and mode after binning scores). FPs could be eliminated for each morphological subclass ( Table S1), with most of the segmentation filters acting on their targeted subclass. However, the effects of each filter were not exclusive to those subclasses.

To evaluate individual filter performance, the percentage of FPs removed by each filter was calculated for the *HC-mixed* image set ( Table 4). A two-sample Kolmogorov–Smirnov test (K–S) was also performed for each filter (α=0.05) on the same data, where one group consisted of the filter scores of all TPs (n=183) and the other group consisted of the scores of all FPs (n=158). All 8 filters rejected the null hypothesis ( Table 4), suggesting that these groups are distinguishable by thresholded segmentation filters. Applying the *intercandidate contour symmetry* filter (filter viii) achieved the largest overall reduction of FPs (44.9%), and eliminated the most SCS-induced FPs (43 of 51) in the low dose exposure set of metaphase images ( Table S1). The *max width* filter (filter iv) yielded the next largest reduction in FPs (27.8%) and was the most efficient filter for detecting the fragmented chromosome class of FPs (8 of 10).

**Table 4.** Comparison of false positive (FP) discrimination ability between proposed DC filters.

DC filter designation **	2-sample K–S, TPs/FPs, p-value *	FP removed (%) *
i: Area	2.2E-18	22.2
ii: Mean width	9.2E-10	16.5
iii: Median width	3.3E-9	14.6
iv: Max width	3.3E-8	27.8
v: Centromere width	8.8E-3	13.9
vi: Oblongness	1.1E-24	27.2
vii: Contour symmetry	1.2E-8	10.1
viii: Intercandidate contour symmetry	4.0E-30	44.9

*Calculated from *HC-mixed* image set from Table 1.

**See Methods section 2 for description of each filter.

FPs were eliminated cumulatively by combining multiple segmentation filters. Since individual filters were separately thresholded to avoid removal of TPs, the inclusive disjunction (logical “or” operation) of multiple filters produced a stronger FP discriminator, but was not expected to reduce the TP count. Different combinations of filters were tested using forward selection ( Table S2). The best performing filter set removed 58.9% of FPs and consisted of 5 FP filters (i + iv + v + vi + viii). Of these, iv and viii accounted for 54.4% of the FPs, with the others identifying the remaining FPs. Performance was evaluated with independent sets of metaphase images ( Table 5), consisting of two HC image sets at low and high dose exposures ( *HC-low* and *HC-high*) and one CNL image set exposed to low dose radiation ( *CNL-low*). On average, 55 ± 9.6% of FPs were removed among all sets; individually, the filters eliminated 52% FPs from *CNL-low*, 66% from *HC-low,* and 48% FPs from *HC-high*. All TPs were retained in each of the sets after FP filtering (i.e. 100% specificity).

**Table 5.** Performance evaluation of false positive (FP) filters * on development and validation image datasets.

Image set **	No. of TP DCs removed	No. of FP DCs removed	FP removed (%)
*HC-low*	0	64	66
*HC-high*	0	29	48
*CNL-low*	0	43	52

*FP filters refer to the subset of filters i + iv + v + vi + viii (Methods section 2).

**See Table 1 for sample details.

**Table 6.** Dose estimation of test samples, with and without false positive (FP) filters * enabled.

	HC samples **			CNL samples **		
Physical dose (Gy)	3.1	2.3	1.4	1.8	2.8	3.4
Estimate, unfiltered (Gy)	3.95	1.70	0.40	1.40	2.45	2.95
Estimate, FP filters (Gy)	2.65	1.4	0.20	2.20	2.80	3.50

*FP filters refer to the subset of filters i + iv + v + vi + viii (Methods section 2). Calibration curve image data were not curated or filtered. HC samples were unselected (INTC03S01: 540 images, INTC03S08: 637 images, and INTC03S10: 708 images) and The CNL samples were previously manually selected (INTC03S04: 448 images; INTC03S05: 500 images; INTC03S07: 385 images).

**See Table 3 for sample details.

**Figure 4.**
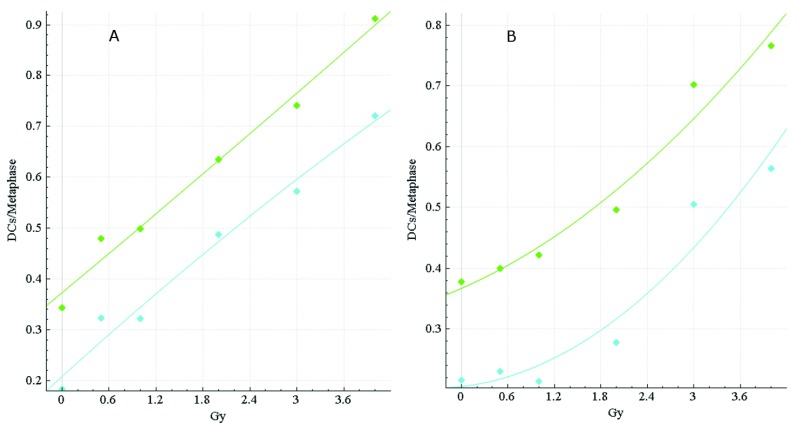
Calibration curves for HC and CNL samples. The dose-response calibration curves for ( **A**) HC and ( **B**) CNL metaphase cell image sample data. Response (mean DC frequency/cell) is on the vertical axis, corresponding radiation dose (Gy) on the horizontal axis. Green curves are based on unfiltered images, cyan curves were derived by recomputing DC frequencies after applying false positive (FP) filters (filters i + iv + v + vi + viii). HC and CNL curves were constructed by fitting a linear-quadratic curve through their respective HC and CNL calibration samples (refer to Table 2). The CNL curves consistently showed a more pronounced quadratic component than the HC curves, which exhibited a nearly linear response. The curves before (green) and after applying FP filters (cyan) are shown. After application of the filters, the HC and CNL curves showed diminished response at different Gy levels, due to elimination of some FP DCs.

Dose calibration curves for HC and CNL data were generated in ADCI to investigate the impact of the FP filters on dose estimation accuracy ( Figure 4). Dose estimation errors, the absolute difference between dose estimate by ADCI and the known physical dose, were determined for three CNL and three HC test samples from HC; then, results for uncorrected vs. FP-filtered images were compared ( Table 6). In manually curated samples from CNL, accuracy was also improved >2-fold by applying the FP filters (average error decreased from 0.43 Gy to 0.18 Gy).

Surprisingly, the dose accuracy of the HC samples did not improve after application of the FP filters (mean absolute error increased from 0.85 Gy to 1.03 Gy). All objects eliminated with these filters in the three HC test samples were reviewed and manually classified as either TP or FP, and the FP specificity across the samples was determined ( Table 7), where FP specificity was defined as the ratio of FPs to all filtered objects. Similar to our earlier findings, the FP filters exhibited very high specificity for FPs (97.7–100%), indicating that the filters retained high specificity for TPs in the HC samples.

**Table 7.** Specificity of false positive (FP) filters * in HC test samples.

Image sample **	Total no. of chromosomes removed	No. of TPs removed	No. of FPs removed	Specificity for FPs
INTC03S01	193	0	193	100%
INTC03S08	133	3	130	97.7%
INTC03S10	143	2	141	98.6%

TP, true positive

*FP filters refer to the subset of filters i + iv + v + vi + viii (Methods #2).

**See Table 3 for sample details.

We hypothesized that a difference in image selection protocols between the two laboratories was responsible for the discrepancies seen in classification performance and dose estimation accuracy. CNL manually selected for images deemed suitable for DCA analysis, and HC image selection was done with an automated metaphase classifier that effectively eliminates only images that lack metaphase cells. Manual review of images in these HC and CNL samples suggested differences in input image quality due to these image selection protocols. In concordance with findings from our previous study ^1^, CNL data contained more images with well-spread, minimally-overlapping chromosomes, and fewer images with extreme SCS and chromosome fragments. The HC data contained a greater percentage of high-band-level (less condensed) chromosomes, characteristic of prometaphase/early-metaphase cell images. These chromosomes were the source of many unfiltered FPs, due to the lack of a strong primary constriction at the centromere which affects automated chromosome classification ^15^.

**Figure 5.**
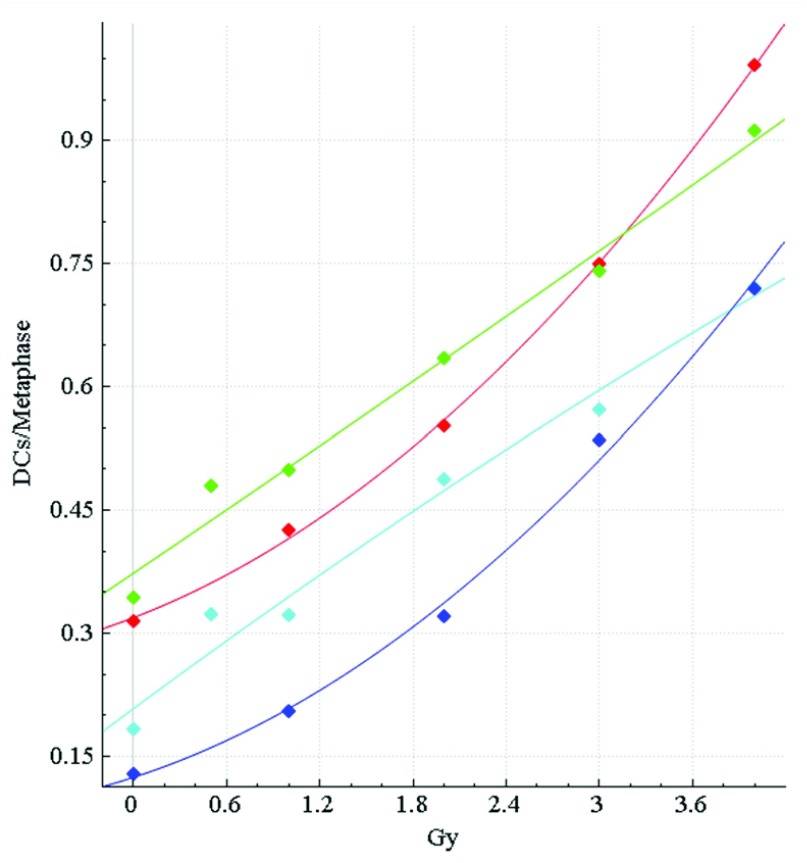
Original vs. manually curated calibration curves for HC samples. The dose-response calibration curves for HC sample data, with and without false positive (FP) filters applied, before and after curation. Response (mean DC frequency/cell) on vertical axis, corresponding radiation dose (Gy) on horizontal axis. Green curve is not curated and includes all images, cyan curve is not curated and applies FP DC filters, red curve is curated, but unfiltered, and dark blue curve is curated and FP filters have been applied. Uncurated curves were generated from 0, 0.5, 1, 2, 3 and 4Gy calibration image data ( Table 2). Curated curves were generated from the same data (except 0.5Gy was excluded) after lower quality images were manually removed (Methods section 4). After manual curation, the HC curves show a stronger quadratic component, similar to the original manually curated CNL curves ( Figure 4).

A new set of HC calibration curves were then generated from manually curated, selected images from calibration samples ( Figure 5). Images were excluded based on IAEA criteria ^17^, along with cells exhibiting long chromosomes in early prometaphase ^16^. Dose estimation accuracy of the HC test samples was significantly improved by enabling the FP segmentation filters (mean absolute error on unfiltered, curated images was 0.37 Gy prior to and 0.15 Gy after filtering; Table 8). Application of FP filters to both CNL and curated HC data led to >2-fold reduction in the mean absolute error of the estimated dose ( *p* = 0.024, paired two tailed t-test). These results motivated the development of approaches to automatically select higher quality metaphase cell images.

**Table 8.** Dose estimates and deviations from physical dose for HC test samples after applying image selection models.

Image selection model	INTC03S01 3.1 *	INTC03S08 2.3	INTC03S10 1.4	INTC03S04 1.8	INTC03S05 2.8	INTC03S07 3.4
All images (unfiltered)	2.65, -0.45	1.3, -1.0	0.2, -1.2	3.2, +1.4	2.1, -0.7	4.5, +1.1
Combined Z Score, weight [5, 2, 4, 3, 4, 1] ^^^, top 250 images	2.85, -0.25	1.8, -0.5	1.05, -0.35	4.65, +2.85	2.5, -0.3	5, +1.6, out of bounds
Combined Z Score, weight [4, 3, 4, 5, 2, 1], top 250	3.0, -0.1	1.8, -0.5	0.95, -0.45	4.95, +3.15	2.35, -0.45	5, +1.6, out of bounds
Combined Z Score, weight [1, 2, 1, 5, 1, 5], top 250	1.55, -1.55	1.35, -0.95	0.4, -1.0	4.4, +2.6	2.7, -0.1	5, +1.6, out of bounds
Chromosome group bin method ^^^, top 250	2.6, -0.5	2.3, +0.0	1.05, -0.35	1.6, -0.2	2.8, +0.0	2.2, -1.2
Filters I-VI	2.05, -1.05	0.95, -1.35	0.35, -0.95	0.6, -1.2	2.05, -0.75	1.15, -2.25
Filters I-III & chromosome group bin method, top 250	3.0, -0.1	2.0, -0.3	0.95, -0.45	1.85, +0.05	2.95, +0.15	2.35, -1.05
Manual image curation	2.85. -0.25	2.3, +0.0	1.05, -0.35	3.95, +2.15	2.45, -0.35	4.0, +0.6

**Figure 6.**
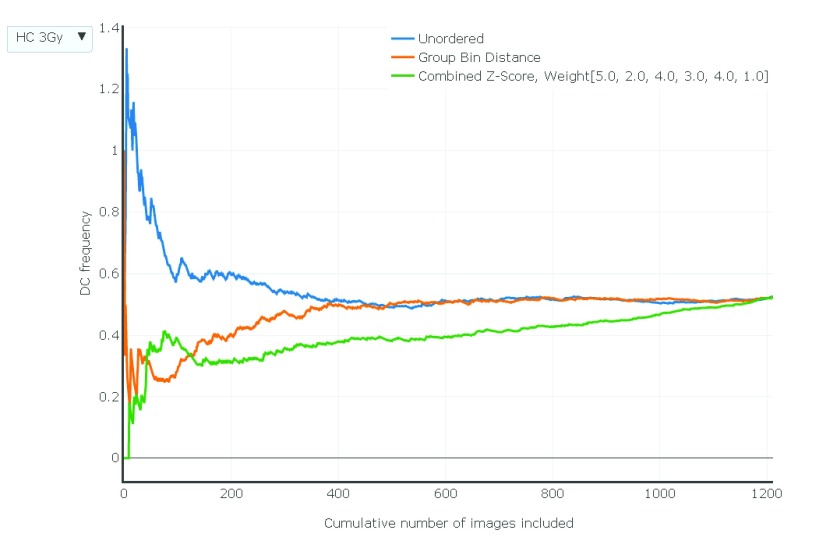
Relationship between DC frequency (y-axis) and number of images used (x-axis) to calculate frequency. Samples exposed to different radiation levels and generated by each laboratory can be toggled and compared using the drop down menu (top left). The static image in the portable document format displays this relationship for the HC sample exposed at 3 Gy. Images were ranked by different scoring methods (see key). DC frequencies based on unordered, unselected images (order corresponds to the alphabetized file names, which is random with respect to image quality) are indicated with a blue line, images ranked by Group Bin Distance are shown in orange, and those ranked according to Combined Z Score are shown in green. Lowest count numbers in the ranked images correspond to the highest quality and lower quality images are progressively introduced as the count increases. Graphs were generated with Plotly ( https://plot.ly/).

*Sample identifier, physical dose (Gy). False positive filters were enabled. Out of bounds indicates that the estimated dose exceeded the maximum calibrated dose. ^ Figure 6 indicates effect of these image selection models on DC frequency for INTC03S04, INTC03S05, INTC03S7 as a function of the number of images analyzed.

### Application of image selection models

To the best of our knowledge, assessment of metaphase cell image quality for DC analysis has not been objectively and quantitatively standardized between laboratories. Cell selection by cytogenetic experts is based on their knowledge of metaphase chromosome conformation, sensitivity, and even individual preferences in interpreting images that can sometimes be inconsistent. Therefore, image selection methods were evaluated through dose estimation of filtered test samples and comparisons with known physical exposures. Images in all calibration and test samples from the same laboratory were processed by the same image selection model. Dose estimates of test samples were calculated using a curve fit to the dose-response of calibration samples. Dose estimation errors indicate the accuracy of dicentric chromosome detection, and therefore provide a means of assessing the accuracy based on the image selection model used.

Each image in a sample was ranked based on its Combined Z Score, which is the sum of the products of the Z score for each of the filters (I – VI) and their corresponding weights. Weights were assigned integer values from 1 to 5. The optimal weights were obtained by searching all possible integer values among the set of calibration samples to determine those exhibiting smallest residual differences with the physical dose after fitting these estimated doses to the curve. This approach, while limiting the search space and reducing the computational complexity, ensured that a diverse combination of weights were used to evaluate each sample. The three optimal weight vectors resulting from this analysis, [5, 2, 4, 3, 4, 1], [4, 3, 4, 5, 2, 1], and [1, 2, 1, 5, 1, 5], were used to independently estimate doses of test samples of unknown exposure.

After images from a sample were assigned either Combined Z Scores or Group Bin Scores and sorted by rank, the 250 top ranked images were selected to determine dicentric aberration frequency for that sample. An adequate number of top ranked images are selected to provide sufficient images to generate a reproducible DC frequency for that sample. In the absence of a predicate filtering step, the ranking procedure has to effectively remove poor quality images that could distort the DC frequency. IAEA recommends >100 DCs be counted for samples with physical doses < 1 Gy ^17^. In practice, laboratories usually score >250 images, but at least 500 cells may be required to achieve this level of DC detection and often more are required for samples with low radiation exposures. Selecting at least the 250–300 top scoring images resulted in stable dicentric frequencies for samples from both laboratories over a range of exposures ( Figure 6: the interactive version allows viewing of individual calibration samples from 0 to 4 Gy exposure and three blinded samples from both the CNL and HC laboratories; the HC3Gy sample is shown in the static PDF version). Compared to the unselected, unordered images, the image selection models show a monotonic increase of DC frequency with radiation dose for image counts with stable frequencies for most samples (eg. HC2Gy, HC3Gy, HC-INTC03S10, CNL2Gy, CNL INTC03S05, CNL-INTC03S07). However, DC frequencies can differ by image selection method. For higher ranking images, the Combined Z Score more consistently eliminated cells with DCs than the Group Bin Distance Scoring method, resulting slightly lower overall DC frequencies, which may be due to more stringent selection of cells possessing fewer FPs. Dose responses for the image selection methods are generally lower for samples with large numbers of top ranked, high quality images, which gradually increase with lower image quality due to the presence of increasing numbers of unfiltered FP DCs. By contrast, unfiltered randomly sampled images from the same sample exhibit higher overall DC frequencies due to increased numbers of FP DCs. As expected, all of the DC frequencies converge to the same value when none of the images are excluded by the ranking methods.

Deviations of the estimated doses of the HC and CNL test samples from their corresponding physical exposures were determined for various image selection models ( Table 8 and Table 9, respectively). For comparison, the dose estimation results of unselected, comprehensive sets of images for each sample are also shown. Deviations of ≤ 0.5 Gy from their calibrated physical dose are considered acceptable in triage biodosimetry ^5, 12, 17^. For the unfiltered HC samples, the average absolute error was 0.8 Gy, and only a single sample, INTC03S01, fulfilled the triage criteria. The image selection model comprising filters I-III sorted by Chromosome Group Bin rank was the most accurate, with dose estimates for 4 HC samples (INTC03S01, INTC03S08, INTC03S10 and INTC03S05) exhibiting acceptable error tolerances (± 0.5 Gy). The Combined Z Score ranking with weights: [1, 2, 1, 5, 1, 5] had the lowest dose estimation accuracy for the HC samples (average error is ~1 Gy), with only INTC03S05 having an acceptable dose estimate. Of the 5 unfiltered, manually curated CNL samples, only INTC03S08 had an acceptable dose estimate. However, an image selection model consisting of all Z Score filters I-VI was the most accurate for CNL samples (mean absolute error of ~0.3 Gy), with 4 of 5 samples (INTC03S08, INTC03S04, INTC03S05 and INTC03S07) having acceptable estimated doses.

**Table 9.** Dose estimates and deviations from physical dose for CNL test samples after applying image selection models.

Image selection model	INTC03S01 3.1 *	INTC03S08 2.3	INTC03S04 1.8	INTC03S05 2.8	INTC03S07 3.4
All images	4, +0.9, out of bounds	2.6, +0.3	2.45, +0.65	3.6, +0.8	4, +0.6, out of bounds
Combined Z Score, weight [5, 2, 4, 3, 4, 1] ^^^, top 250	3.9, +0.8	2.8, +0.5	2.1, +0.3	3.05, +0.25	3.55, +0.15
Combined Z Score, weight [4, 3, 4, 5, 2, 1], top 250	4, +0.9, out of bounds	2.75, +0.45	1.7, -0.1	3.05, +0.25	3.9, +0.5
Combined Z Score, weight [1, 2, 1, 5, 1, 5], top 250	3.6, +0.5	2.4, +0.1	0.65, -1.15	2.35, -0.45	3.05, -0.35
Chromosome group bin method ^^^, top 250	4, +0.9, out of bounds	2.8, +0.5	1.8, +0.0	2.55, -0.25	4, +0.6
Filters I-VI	3.75, +0.65	2.8, +0.5	1.95, +0.15	3.05, +0.25	3.4, +0.0
Filters I-III & chromosome group bin method, top 250	4, +0.9, out of bounds	2.75, +0.45	1.65, -0.15	2.25, -0.55	3.95, +0.55
Manual image curation	3.7, +0.6	2.85, +0.55	2.2, +0.4	2.8, +0.0	3.5, +0.1

*Sample identifier, physical dose (Gy). False positive filters were enabled. Out of bounds indicates that the estimated dose exceeded the maximum calibrated dose. ^ Figure 6 indicates the effect of these image selection models on DC frequency for INTC03S01, INTC03S08, INTC03S10 as a function of the number of images analyzed.

While automated image selection rejects poor images and reduces FP DCs, dose estimates can only be considered reliable if sufficient numbers of images remain after filtering. Application of image filters can result in fewer than the recommended images for accurate dose estimation. Samples CNL-INTC03S08 and HC-INTC03S07, had 195 and 109 metaphase cells, respectively, after filtering and image selection. HC-INTC03S07 was of relatively lower quality, and the unfiltered set of 477 metaphase images contained fewer than the recommended minimum number after filtering ( Table 10).

**Table 10.** Goodness of fit Poisson scores* of unfiltered, manually- and ADCI-filtered calibration and test samples.

Sample	Unfiltered images	Manual image selection	Automated image selection
HC0Gy	1.3E-15	2.2E-01	No DCs detected
HC05Gy	4.1E-32	Unavailable	1.1E-02 ^^^
HC1Gy	1.6E-18	9.9E-01	1.0E-01 ^^^
HC2Gy	4.0E-64	2.1E-01	7.6E-04 ^^^
HC3Gy	2.9E-02	4.6E-01	6.1E-01 ^^^
HC4Gy	2.6E-04	2.2E-01	3.1E-01 ^^^
HC-INTC03S01	<2.2E-308 ^+^	9.1E-01	1.2E-01 ^^^
HC-INTC03S08	1.2E-01	4.6E-01	8.2E-01 ^^^
HC-INTC03S10	8.9E-01	3.9E-01	2.1E-01 ^^^
HC-INTC03S04	<2.2E-308 ^+^	<2.2E-308 ^+^	5.5E-01 ^^^
HC-INTC03S05	1.1E-06	7.3E-05	3.5E-03 ^^^
HC-INTC03S07	<2.2E-308 ^+^	<2.2E-308 ^+^	2.0E-04 ^^^
CNL0Gy	5.2E-03	1.3E-01	3.1E-01 ^#^
CNL05Gy	1.7E-157	1.2E-01	6.0E-32 ^#^
CNL1Gy	9.8E-30	1.5E-03	1.6E-06 ^#^
CNL2Gy	2.3E-147	<2.2E-308 ^+^	4.5E-02 ^#^
CNL3Gy	8.5E-07	6.8E-03	9.9E-01 ^#^
CNL4Gy	5.2E-22	3.3E-02	1.8E-01 ^#^
CNL-INTC03S04	1.7E-60	1.9E-02	5.4E-02 ^#^
CNL-INTC03S05	2.7E-09	5.2E-02	3.3E-01 ^#^
CNL-INTC03S07	6.7E-10	4.2E-05	4.7E-01 ^#^
CNL-INTC03S01	<2.2E-308 ^+^	3.7E-03	7.6E-11 ^#^
CNL-INTC03S08	5.3E-16	7.7E-01	7.8E-01 ^#^

*Poisson score is the p-value of chi-square goodness of fit (without merging bins) of observed distribution of DCs/cell vs. Poisson distribution determined from average DC frequency. Filtering parameters chosen for each laboratory exhibit dose estimates that are closest to the physical dose:

^HC image sets were selected with filters I-III and ranked by chromosome group bin score;

^+^Minimum positive floating value in Windows operating system;

^#^CNL image sets were selected with filters I-VI.

### Sample quality assessment after image selection

To determine if image selection improved sample quality, a Chi-squared goodness of fit test on Poisson distributed DCs was performed, both before and after automated and manual image selection ( Table 10). Manual image selection for CNL samples was performed by CNL during sample preparation, while image selection for HC samples was performed on unselected datasets (samples HC-INTC03S01, HC-INTC03S08, HC-INTC03S10 were analyzed, despite <500 available images). The optimal image selection models were used for FP and image filtering for each laboratory ( Table 8 and Table 9). The HC samples were selected with filters I-III and the chromosome group bin method, whereas the CNL samples were processed with filters I-VI. At the 1% significance level (i.e. Chi-square goodness-of-fit, p ≤ 0.01), 86% (19 of 22) of unfiltered samples were significantly different from the Poisson distribution, and 76% (13 of 17) of manually- and 77% (17 of 22) of automatically-selected samples did not differ. Manually curated and uncurated sample groups also significantly differed from each other (p = 0.0021; one-tailed Wilcoxon Signed-Rank Test, α=0.05, n=17). Therefore, the Poisson goodness of fit measures improvements in overall sample quality from image model selection. While the overall goodness of fit is improved for all of the automatically selected datasets, the Poisson distributions of DCs in the lowest quality samples (CNL1Gy, CNL05Gy, CNL-INTC03S01, HC-INTC03S05, HC-INTC03S07) were still rejected at a 0.5% significance threshold after filtering.

## Discussion

Automated biodosimetric methods to detect DCs can produce incorrect assignments because the algorithms cannot capture the full range of morphological variability inherent in chromosome images of metaphase cells. Accuracy of these radiation exposure estimates can be improved by morphology-based chromosome image segmentation filters that eliminate suboptimal metaphase cell images and false positive DCs in the remaining images. Compared to results generated by the previous version of ADCI which did not reclassify FPs or remove any cell images ^11^, the filters described here reduced FP DC rates by ~55% across a wide range of radiation exposure levels. Additionally, we showed that the object segmentation filters were highly specific for FPs in test image sets consisting of irradiated samples blinded to known dose (97.7–100%, n=6). Overall, the FP filters substantially improved DC classification accuracy.

The segmentation filters successfully target the majority of cells with SCS and chromosome fragments. The *intercandidate contour symmetry* filter is a particularly promising SCS detector, individually eliminating 84% of all SCS-induced FPs in our test dataset. Acrocentric chromosomes were disproportionally susceptible to SCS-induced errors compared to other chromosome types (69% of SCS cases, despite making up only 22% of human chromosomes). Given the rarity of acrocentric TP DCs (due to width profile inaccuracies at the extreme ends of chromosomes ^7– 9^), filters targeting acrocentric or small chromosomes, in general (such as filters i and vi), can also be useful for reducing SCS-induced FPs.

Certain FP subclasses were commonly targeted by multiple filters. Redundancy among the segmentation features resulted in only a subset of the filters being required to maximize FP elimination. Notably, filters ii–v eliminated FPs based on different definitions of chromosome width. The final FP filter combination consisted of only 5 of the 8 originally proposed filters. However, it should be noted that a combination of only 2 of the filters - the *intercandidate contour symmetry* (viii) and *max width* (iv) filters - achieved nearly the same level of FP detection in the test sample dataset, with the others having only incremental benefit.

The image selection filters were required to be scale-invariant, since chromosome structures may vary between cells, individual samples, and laboratory preparations. Scale invariance is also necessary to control for pixel-based chromosome measurements affected by chromosome condensation differences within a metaphase cell, and differences arising from optical magnification. This was achieved by either using image level filter scores normalized to the median “raw” score of all objects within the same cell image (i.e. filters I–V), or in which scores were determined from the ratios of pixel-based features (i.e. filters VI–VIII).

Differences in accuracy between the manually- and automatically-selected images for dose estimation revealed limitations of the current set of filters. The FP object filters in the manually curated CNL and HC image samples reduced the average dose estimation error from 0.4 Gy to <0.2 Gy (with a maximum error of 0.4 Gy), respectively. However, solely applying the FP object filters to unselected HC metaphase data was insufficient to correct this problem (average error increased by 0.15 Gy), and led to more inaccurate dose response values.

Variability in cell image quality contributed to this source of error. Some unselected HC samples contained images with high levels of SCS, which upon processing, produced large numbers of incorrectly classified chromosome fragments in some cells. While FP DC filters i–v target detection of these fragments, they were not reclassified in these cells, because they comprised the predominant chromosome morphology. For similar reasons, FP filtering was not suitable for elimination for removal of FPs in prometaphase cells containing many high resolution, long chromosomes (>700 band level). These observations suggested the need for another class of morphological filters that operated on complete images to remove those of low quality prior to dose estimation.

Image quality is a critical aspect of accurate DC detection and dose estimation. Manual inspection and quality control of metaphase selection is a common and essential practice in cytogenetic and biodosimetry laboratories, but it can be labor-intensive, and is frequently not automated. Image-level filtering automatically applies statistical thresholds to eliminate chromosomes with morphological features and non-chromosomal objects that predispose to FP DC assignments. Image scoring methods can also select a defined number of top-ranked, processed images for dose estimation. These FP filtering and image scoring methods can be applied either individually or in combination, resulting in improvements in the accuracy of DC frequency. Errors in dose estimates are considerably reduced using suitable image selection models in samples with ≥250 images. Doses were accurately estimated for most test samples within ±0.5Gy of their physical doses, as recommended ^17^. Therefore, the image selection models presented provide reliable quality control, and can minimize manual review or DC analysis.

Automated image selection aims to simulate manual image curation. At this point, it does not quite achieve the same overall accuracy as manual image selection, especially for samples containing numerous images of lower quality. However, the respective differences in dose estimates of higher quality samples from HC and CNL, especially at exposures >2 Gy, are not significant. Automating image selection, nevertheless, offers unique advantages over manual image selection by introducing a uniform approach for chromosome analyses, ensuring both increased reliability and speed.

## Data and software availability

Python code and sample data files for “Accurate cytogenetic biodosimetry through automated dicentric chromosome curation and metaphase cell selection” are available at http://doi.org/10.5281/zenodo.833536 ^18^.

MATLAB code and sample data files for “Accurate cytogenetic biodosimetry through automated dicentric chromosome curation and metaphase cell selection” are available at http://doi.org/10.5281/zenodo.833540 ^19^.

Source code license: CC-BY 4.0

## Supplementary material

Supplementary File 1: File containing *Morphological characterization of FPs*, Figure S1, and Table S1 and Table S2.
