# Supplementary material for: Accurate cytogenetic biodosimetry through automated dicentric chromosome curation and metaphase cell selection
Source: F1000Res. 2017 Aug 9;6:1396. [Version 1] doi: 10.12688/f1000research.12226.1 (PMC5583746; doi:10.12688/f1000research.12226.1)
Supplement: Supplementary file 1 [file f1000research-6-13236-s0000.tgz › 7301f88f-280c-41b4-a20b-26670b44c696.docx]

**SUPPLEMENTARY INFORMATION**

**Morphological characterization of False Positives (FP)**

FPs and TPs from the *HC-low* image set (Table 1) were compared according to their respective segmentation features, including contour, width profile, centerline placement, centromere candidate placement, and total pixel area. FPs (n=98) were grouped by common distinguishing traits and assigned to one or more of the following morphological classes:

1. **Sister chromatid separation:**

Sister chromatid separation (SCS) of a chromosome refers to the loss of sister chromatid cohesion at the telomeres, and often along the sister chromatids, excluding the centromeres. Due to inherent limitations of a centerline derived from contour skeletonization in chromosomes, SCS often resulted in partial or complete localization of the centerline along a single chromatid, rather than along the long axis of the full-width chromosome*^8-10^*. Complete centerline localization to chromatids of the q-arm was common among acrocentric chromosomes (Figure S1A). This resulted in a width profile in which the displaced centerline did not accurately represent the width of the chromosome, and compromised centromere determination.

1. **Chromosome fragmentation:**

Sister chromatid pairs were completely dissociated in metaphase images, resulting in incorrect labeling of each chromatid as separate chromosomes. Occasionally, segmentation fragmented images of intact non-uniform chromosomes into multiple, chromosomal artifacts[*^6^*](#_ENREF_3) (Figure S1B). Artifactual fragmentation into incomplete chromosome fragments led to unpredictable results, increasing FPs and FNs.

1. **Chromosome overlap:**

Poor spatial separation of chromosomes produced clusters of overlapping/touching chromosome clusters which were inseparable. Occasionally, the cluster is segmented as a single contiguous object (Figure S1C). Like chromosome fragments, analysis of these overlapping chromosome clusters produces erroneous results. FP DCs were produced from clusters comprising two underlying monocentric chromosomes, each contributing a centromere to the combined object.

1. **Noisy contour:**

Poor image contrast at the chromosomal boundary produced “noisy,” jagged chromosome contours contributing multiple small constrictions to the width profile (Figure S1D). These artifactual constrictions were incorrectly identified as multiple centromeres if their magnitudes were similar to the true centromere, leading to a FP assignment.

1. **Cellular debris:**

Non-chromosomal objects such as nuclei and cellular debris were generally removed by pre-processing based on thresholding relative size and pixel intensity. However, aggregated cellular debris were occasionally labelled as a chromosome and naively analyzed by the software (Figure S1E).

1. **Machine learning error:**

A “catch-all” subclass for MCs with no identifiable morphological traits and reasonable contours and centerlines (Figure S1F). These cases reflect deficiencies in the feature set or training data of the machine learning (ML) classifiers, rather than image segmentation errors.


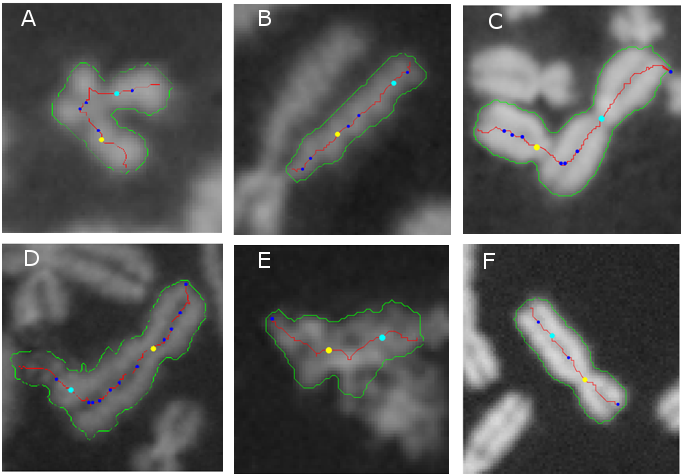


**Figure S1.** Examples of FPs in each morphological subclass. The subclasses are defined in the Methods. Chromosome contours are displayed in green, centerlines in red, top-ranked and 2^nd^-ranked centromere candidates in yellow and cyan, respectively, and other centromere candidates in blue. (A) SCS: An MC with SCS showing the characteristic localization of centerline along chromatid. (B) Chromosome fragment: Artifactual fragmentation of a chromosome caused by overaggressive image segmentation. (C) Chromosome overlap: Two touching MCs treated as a single DC (under-segmentation). (D) Noisy contour: The jagged contour due to poor image contrast is prone to introducing artifactual width constrictions. (E) Cellular debris: Incorrectly processed as a chromosome. (F) ML deficiency: An MC with no notable errors in contour or centerline.

**Table S1. Comparison of FP subclass targeting between proposed DC filters.**

|  | **No. FP DCs removed filtering by morphological subclass*** | | | | | |
| --- | --- | --- | --- | --- | --- | --- |
| **DC filter designation**** | SCS (n=51) | Fragment  (n=10) | Overlap (n=17) | Noise (n=5) | Debris (n=4) | ML (n=11) |
| I: Area | 19 | 6 | 0 | 0 | 3 | 1 |
| II: Mean width | 14 | 6 | 0 | 0 | 2 | 0 |
| III: Median width | 12 | 5 | 0 | 0 | 3 | 0 |
| IV: Max width | 23 | 8 | 0 | 0 | 3 | 0 |
| V: Centromere width | 8 | 3 | 0 | 0 | 3 | 0 |
| VI: Oblongness | 31 | 1 | 2 | 0 | 0 | 0 |
| VII: Contour symmetry | 11 | 0 | 0 | 0 | 0 | 0 |
| VIII: Intercandidate contour symmetry | 43 | 2 | 3 | 1 | 1 | 2 |

*****See above for a description of each subclass. Calculated from the *HC-low* image set in Table 1.

**See Methods for a description of each filter.

**Table S2. Forward selection results by combining subsets of DC filters.**

| **DC filter subset**** | **FP removed (%)*** |
| --- | --- |
| 1-filter: VIII | 44.9 |
| 2-filters: VIII + IV | 54.4 |
| 3-filters: VIII + IV + V | 56.3 |
| 4-filters: VIII + IV + V + VI | 58.2 |
| 5-filters: VIII + IV + V + VI + I | 58.9 |

*Calculated from *HC-mixed* image dataset in Table 1.

**See Methods 2 for description of each filter.
