# Supplementary material for: Accurate cytogenetic biodosimetry through automated dicentric chromosome curation and metaphase cell selection
Source: F1000Res. 2017 Aug 9;6:1396. [Version 1] doi: 10.12688/f1000research.12226.1 (PMC5583746; doi:10.12688/f1000research.12226.1)
Supplement: Peer review history

# Peer review: Referee response for version 1

**DOI:** 10.5256/f1000research.13236.r25106

**Published:** 2017-08-31

**Authors:** Eric Gregoire

**Affiliations:** 1 Biological Dosimetry Laboratory (LDB), Institute for Radiological Protection and Nuclear Safety (IRSN), Fontenay-aux-Roses, France

This paper presents an approach for the improvement of automatic detection of dicentrics and particularly the removal of some kinds of False Positives (FP). Indeed, the dicentric is the standard bio-marker in biological dosimetry. However the manual scoring of dicentric chromosome is time consuming and the improvement of the result time is necessary in biological dosimetry. For dose assessment in case of triage of radiation exposed population, automatization of dicentric detection is very useful.

 In order to increase the dicentric detection, the authors concentrate the study on the removal of FPs as well as on the selection of quality images. Apparently the main FPs in the metaphases of the 2 study laboratories are Sister Chromatid Separation (SCS) (84% of the FPs). This study shows that the authors reach to eliminate them with filters (about 55% of SCS removed). This is great and encouraging to remove other kind of FPs.

 The study showed also the importance of selecting metaphases before the analysis and the detection. The quality of images and chromosomes is an important factor. The dose assessment becomes better when the images were selected than when they are not selected. The data show a difference in dose assessment between the 2 labs. The filters gave better results for CNL images than for HC images particularly on low doses. The dose assessment may be a problem of sampling or of the number of analyzed metaphases. this could be improved.

 However the selection of good quality metaphases is also time-consuming. There is a good comparison between manual and automatic triage of metaphases for the 2 labs highlighted by the statistical tests. On the other hand, the number of metaphases analyzed remains important for the dose estimation as well as the detection of true dicentrics.

 Perhaps it should be interesting to compare this algorithm to the Metafer DCScore algorithm proposed by Metasystems in terms of detected dicentric rate and dose assessment.

 Overall the authors are to be congratulated on a well-presented study of their work.

 Page 7 last paragraph : prominent sister CHROMOSOME separation. Is it right or is it a mistake instead of CHROMATID?

I have read this submission. I believe that I have an appropriate level of expertise to confirm that it is of an acceptable scientific standard.

# Peer review: Referee response for version 1

**DOI:** 10.5256/f1000research.13236.r24918

**Published:** 2017-08-25

**Authors:** David Lloyd

**Affiliations:** 1 Centre for Radiation, Chemical and Environmental Hazards (CRCE), Public Health England, Didcot, UK

This paper presents a considerable amount of work devoted to improving the accuracy of existing automatic dicentric systems for biological dosimetry.  The authors have developed some very interesting ideas in constructing filters that detect false positive dicentrics and thereby improve the accuracy of ‘hands-off’ microscopy. The work has demonstrated that inter-laboratory differences, commonly reported in biological dosimetry, and evident between the two labs here, can be considerably improved by application of the filters. It is particularly interesting to see how the linear dose response reported by one lab, over a dose range that should show a lin/quadratic  curve, can indeed be converted to linear quadratic by filtering out the false positives. Moreover they have demonstrated that much of the improvement can be achieved by a sub-set of their filters which should simplify future developments by not needing to employ all the methods.

 The single most pressing remaining problem is the selection of metaphases of sufficient quality for passing onto the filtration procedures. Manual selection still seems better for removing the wide range of unsuitable material although the authors have demonstrated that the automated approach is getting there. The authors suggest that manual screening of candidate  metaphases is labour intensive but most experienced cytogeneticists would probably consider that  a list of, say, 1000 automatically presented images can be screened by eye in a few minutes.  This is probably acceptable for much routine biological dosimetry but I agree that fully automated image selection would be particularly advantageous when rapid triage dosimetry sorting of many cases is needed.

 It is gratifying to see that the accuracy of dose estimations using the procedures described here falls well within the requirements for triage sorting following a major radiological incident.

 I note that this system uses Giemsa stained material examined with bright-field microscopy. There is an alternative approach being employed in biological dosimetry which uses fluorescent probes to highlight centromeres. I wonder if the authors would like to comment on this and speculate on the extent to which their filtration ideas could be applied to this approach too.

 Overall they are to be congratulated on a well-presented account of an improved approach to automated ‘dicentric-hunting’.

I have read this submission. I believe that I have an appropriate level of expertise to confirm that it is of an acceptable scientific standard.
